# How much money is spent on vaccines across Western European countries?

**DOI:** 10.1080/21645515.2016.1155013

**Published:** 2016-05-25

**Authors:** Olivier Ethgen, Florence Baron-Papillon, Murielle Cornier

**Affiliations:** a Department of Public Health Sciences, Faculty of Medicine, University of Liège, Liège, Belgium; b SERFAN Innovation, Namur, Belgium; c Sanofi Pasteur MSD, Lyon, France

**Keywords:** budget, prevention, spending, vaccines, Western Europe

## Abstract

Prevention programs, particularly vaccinations, remain highly vulnerable to budget cuts because their benefits may not be immediately identifiable. Seven Western European countries were selected (Germany, England, France, Italy, Spain, Sweden and Portugal) constituting a good mix of vaccine procurement modalities, with the objective to document the proportion of healthcare spending devoted to vaccines and its evolution. A data search was performed using the OECD online databases and official national sources from 2008 (2006 for England). No country spent more than 0.5% of its healthcare budget on vaccines. The proportion ranged from 0.25% in Spain (2012) and France (2013) to 0.47% in Germany (2014). Whereas healthcare spending increased in all countries but Spain (with increases ranging from +2.6% per year in France between 2008 and 2013 to +8.1% per year in England between 2006/07 and 2009/10), vaccine spending diminished markedly in Germany (−6.2% per year from 2008 to 2014), Spain (−6.7% per year from 2008 to 2012) and France (−4.2% per year from 2008 to 2013). Only Sweden (+5.9% per year from 2011 to 2013) and England (+18.9% per year from 2006/07 to 2009/10) increased their spending on vaccines. Vaccination involves relatively low levels of healthcare investment in Western Europe relative to the far-reaching public health benefits that it provides. We found a net trend toward a decrease in such spending in recent years, with the exception of Sweden and England. Vaccination budgets should be preserved or even increased to sustain a life-course approach to immunization with sufficient coverage rates.

## Introduction

The history of vaccination began more than 2 centuries ago with the discovery of the smallpox vaccine. Further discoveries followed throughout the 20th century, and 25 communicable diseases (10 bacterial diseases and 15 viral diseases) can currently be prevented by vaccines.^1^ Similar to many other developed countries, European countries have established immunization schedules to protect their populations against the threat of infectious diseases.^2^

The implementation of an immunization schedule that protects people from approximately 20 infectious diseases requires appropriately dedicated resources. Vaccine purchase and administration as well as logistics by healthcare professionals mobilize resources every year.^3^ Over the last 2 decades, policy makers have paid increasing attention to the healthcare costs in view of reduced budgetary capacity.

Although prevention accounts for a minor part (less than 5%) of the healthcare spending in most of Western Europe, prevention programs, particularly vaccinations, remain highly vulnerable to budget cuts because their benefits may not be immediately identifiable.^4,5^ This study thus aimed to document the proportion of national healthcare budgets spent on prevention and, more specifically, on vaccination across Western European countries.

## Results

### Vaccine spending

**Figure 1.**
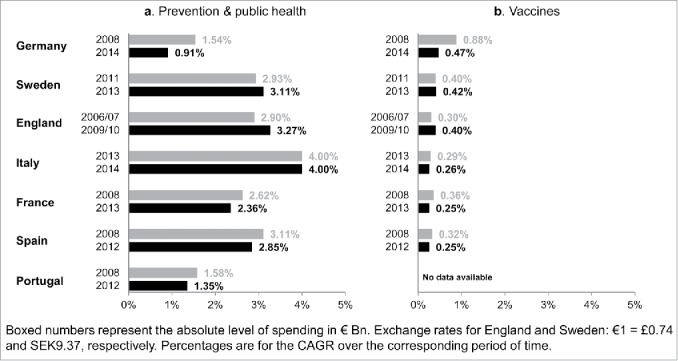
Proportion of national healthcare spending devoted to prevention and to vaccines (national sources).

Figure 1 shows the proportion of the healthcare budget spent on prevention and on vaccine procurement. Based on the latest data point available, the proportion spent on prevention and public health ranged from 0.91% in Germany for 2014 to 4.00% in Italy for 2014 (Fig. 1a).

No country spent more than 0.5% of its healthcare budget on vaccines (Fig. 1b). The proportion spent on vaccines ranged from 0.47% (€ 0.91/194 Bn; €13 per capita) in Germany for 2014 to 0.25% in Spain (€ 0.17/69 Bn; €4 per capita) for 2012 and France (€ 0.63/248 Bn; €10 per capita) for 2013. Sweden spent 0.42% (€ 0.19/46 Bn; €20 per capita) of its healthcare budget on vaccines in 2013, England 0.40% (€ 0.54/136 Bn; €10 per capita) in 2009/10 and Italy 0.26% (€ 0.29/113 Bn; €5 per capita).

Overall, the results revealed a trend toward a diminishing proportion of the healthcare budget spent on vaccines procurement, with the exception of Sweden and England. In France and Spain, this proportion declined from 0.36% and 0.32% in 2008, respectively, to 0.25% in 2012 for Spain and 0.25% in 2013 for France. A more marked decline occurred in Germany, from 0.88% in 2008 down to 0.47% in 2014 (Fig. 1b). In Italy, the decline was from 0.29% to 0.26% but with data only available for 2013 and 2014.

**Figure 2.**
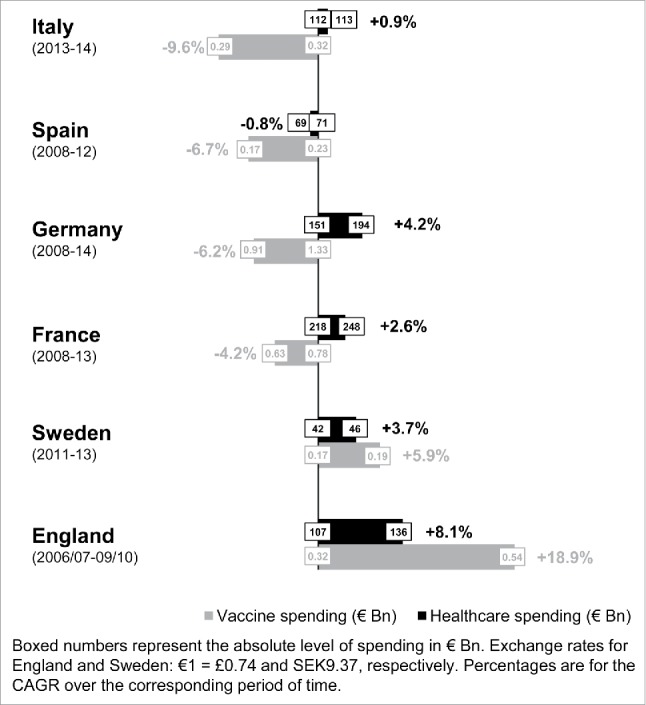
Healthcare and vaccine spending evolution (national sources).

Figure 2 compares the evolution of healthcare and vaccine spending from 2008 to the last data point available. Healthcare spending has increased in all countries but Spain. Increases (expressed in actuarial terms using the CAGR) ranged from +0.9% per year in Italy (but with data only available for 2013 and 2014) to +8.1% per year in England from 2006/07 to 2009/2010. Meanwhile, vaccine spending diminished markedly in Italy (−9.6%, but with data only available for 2013 and 2014), Spain (−6.7% per year from 2008 to 2012), Germany (−6.2% per year from 2008 to 2014) and France (−4.2% per year from 2008 to 2013). Only Sweden (+5.9% per year from 2011 to 2013) and England (+18.9% per year from 2006/07 to 2009/10) increased their vaccine spending.

The data accessible for Portugal concerned only vaccine expenditures in ambulatory markets, while most vaccines are centrally purchased via tender procedures. As such, these data were not considered fully representative of the actual vaccine spending in Portugal and were not included in Figs. 1 and 2. When available, OECD data exhibited similar proportions and evolution patterns on prevention spending for most countries, except for Germany and Italy. Data on prevention of communicable diseases spending were scarce (Table 1).

**Table 3.** National data sources.

	Prevention and vaccine expenditures	National vaccination calendar
Germany	Bundesministerium für Gesundheit (BMG)^1^	Robert Koch Institut - STIKO-Impfkalender 2014
England	Health England^2^	National Health Service (NHS) - summer 2014
	Department of Health^3^	
France	Direction de la Recherche, des Etudes, de l'Evaluation et des Statistiques (DREES)	Ministère des Affaires Sociales et de la Santé (MASS) - 2014
Italy	European House - Ambrosetti Meridiano Sanita^4^	Italian Minitry of Health - PNPV 2012–2014
	Agenzia Italiana del Farmaco (AIFA) - Rapporto OsMed 2014	
Spain	Ministerio de Sanidad, Servicios Sociales e Igualdad (MSSSI)^5^	Ministerio de Sanidad, Servicios Sociales e Igualdad (MSSSI) - 2015
	Suplemento de La Razon - A tu salud (Mayo 2013)	
Sweden	Statistiska Centralbyran (SCB)^6^	Folkhälsomyndighten - 2015
Portugal	Instituto Nacional de Estatistica (INE)^7^	Ministerio da Saude - 2015
		

1 Expenditure from Statutory Health Insurance only (SHI).

2 Data for vaccines expenditure for the year 2006/07 only.

3 Data for vaccine expenditure for the year 2009/2010 only. Prevention expenditure from 2006 to 2011 Program budgeting data.

4 Data for the year 2013 only.

5 Data until 2012 only.

6 Data on immunization programmes for 2011, 2012 and 2013 only.

7 Prevention data for 2008 and 2012, no data on vaccine.

**Table 4.** Comparative overview of prevention spending in OECD and national data sources.

OECD	Germany	England	France	Italy	Spain	Sweden^1^	Portugal
Maternal and child health, family planning and counselling School health services Prevention of communicable diseases (including vaccines) Prevention of non-communicable diseases Occupational health care All other miscellaneous public health services	Social services Mutual aid Support in case of medication errors Vaccinations Prevention of non-communicable diseases Occupational health care Primary prevention in settings other than the workplace Dental prevention	Healthy individuals • Prevention program • Mental health prevention • Other Infectious diseases (other than HIV & AIDS)	Individual prevention • Vaccines • Family planning • Occupational health care • School health services • Tumor screening • Screening-Combating infectious diseases • Other pathologies • Medical examinations • Dental checkups Collective prevention • Campaign for vaccination • Combating addictions • Information, promotion and health education • Environmental hygiene • Occupational hazard prevention • Prevention and combating pollution • Observation, search, regulation • Urgencies and crises • Food safety	Overall prevention spending Vaccine spending - Pneumococcal - Hexavalent - Influenza - Anti-HPV - Others	Maternal and child health, family planning and counselling School health services Prevention of communicable diseases (including vaccines) Prevention of non-communicable diseases Occupational health care All other miscellaneous public health services	Information, education and counselling programs Immunization programs Healthy condition monitoring programs Epidemiological surveillance and risk and disease control	Maternal and child health, family planning and counselling Public health programs (consultations and screenings) and compulsory vaccination, i.e., vaccinations included in the national vaccination calendar Disease prevention (mandatory or non-declaration) Occupational health care

1 Exact content of “immunization programs” could not be found.

**Table 1.** OECD data for prevention spending (no prevention data available for England and the UK).

	Germany (€M)					France (€M)					Italy (€M)				
OECD data	2008	%	2012	%	*CAGR*	2008	%	2012	%	*CAGR*	2008	%	2013	%	*CAGR*
HCTOT: Total current expenditure	255,483		290,421		*3.3*%	202,673		226,775		*2.8*%	134,696		134,884		*0.0*%
HC6: Prevention and public health services	9,424	*3.7*%	9,594	*3.3*%	*0.4*%	4,404	*2.2*%	4,588	*2.0*%	*1.0*%	621	*0.5*%	671	*0.5*%	*1.6*%
HC63: Prevention of communicable diseases	NA	―	NA	―	―	307	*0.2*%	306	*0.1*%	−*0.1*%	NA	―	NA	―	―
	Spain (€M)					Sweden (SEKM)					Portugal (€M)				
OECD data	2008	%	2011	%	*CAGR*	2008	%	2012	%	*CAGR*	2008	%	2011	%	*CAGR*
HCTOT: Total current expenditure	93,899		96,886		*1.0*%	281,968		321,802		*3.4*%	16,603		16,537		−*0.1*%
HC6: Prevention and public health services	2,244	*2.4*%	2,125	*2.2*%	−*1.8*%	10,205	*3.6*%	12,625	*3.9*%	*5.5*%	306	*1.8*%	349	*2.1*%	*4.5*%
HC63: Prevention of communicable diseases	4	*0.0*%	5	*0.0*%	*7.7*%	245	*0.1*%	285	*0.1*%	*3.9*%	NA	―	NA	―	―

### Vaccine schedule and diseases prevented

Table 2 details the diseases prevented according to the national vaccination calendars, distinguishing between healthy individuals and those with underlying conditions in the 7 selected countries at the time of the study. Up to 19 diseases are included in the Western European vaccination calendars. The German schedule comprises up to 17 diseases, and the Swedish schedule includes up to 12 diseases.

**Table 2.** Diseases prevented according to national vaccination calendars (2014 or 2015).

	Germany		England		France		Italy		Spain		Sweden		Portugal	
	*Healthy*	*UC*	*Healthy*	*UC*	*Healthy*	*UC*	*Healthy*	*UC*	*Healthy*	*UC*	*Healthy*	*UC*	*Healthy*	*UC*
Diphtheria	✓	✓	✓	✓	✓	✓	✓	✓	✓	✓	✓	✓	✓	✓
Tetanus	✓	✓	✓	✓	✓	✓	✓	✓	✓	✓	✓	✓	✓	✓
Poliomyelitis	✓	✓	✓	✓	✓	✓	✓	✓	✓	✓	✓	✓	✓	✓
Pertussis	✓	✓	✓	✓	✓	✓	✓	✓	✓	✓	✓	✓	✓	✓
Hemophilius influenza B	✓	✓	✓	✓	✓	✓	✓	✓	✓	✓	✓	✓	✓	✓
Influenza	✓^1^	✓	✓^^3^^	✓	✓^^5^^	✓	✓^^5^^	✓	✓^^5^^	✓			✓^^5^^	✓
Pneumococcal	✓	✓	✓	✓	✓	✓	✓	✓	✓	✓	✓	✓	✓	✓
Meningococcal C	✓		✓	✓	✓	✓	✓	✓	✓	✓			✓	✓
Meningococcal ACWY		✓												
Measle	✓	✓	✓	✓	✓	✓	✓	✓	✓	✓	✓	✓	✓	✓
Mumps	✓	✓	✓	✓	✓	✓	✓	✓	✓	✓	✓	✓	✓	✓
Rubella	✓	✓	✓	✓	✓	✓	✓	✓	✓	✓	✓	✓	✓	✓
Varicella	✓	✓				✓		✓		✓				
Hepatitis A		✓				✓		✓						✓
Hepatitis B	✓	✓		✓	✓	✓	✓	✓		✓		✓	✓	✓
Rotavirus	✓	✓	✓	✓										
Human Papillomavirus^4^	✓	✓	✓	✓	✓	✓	✓	✓	✓	✓	✓	✓	✓	✓
Zoster			✓^2^	✓^2^										
Tuberculosis						✓						✓	✓	✓
Tick-borne encephalitis		✓												

UC: individuals with underlying conditions

1 ≥ 60 y only;

2 70y only;

3 2–4 y and ≥ 65 y only;

4 Girls only;

5 ≥ 65 y only.

At the national level, all countries systematically recommend childhood vaccination against diphtheria, tetanus, poliomyelitis, pertussis, hemophilus influenza B, measles, mumps, rubella and pneumococcal diseases in healthy individuals. HPV vaccination is also recommended across all countries for teenage girls. Apart from Sweden, all countries recommend vaccination against meningococcal C and against influenza for the elderly beginning at 65 years of age (except in Germany, where influenza vaccination is recommended for those 60 and older). Other vaccines are recommended more sparingly. In individuals with underlying conditions, Hepatitis B vaccine is additionally recommended in every country, although recommendations for other vaccinations are more heterogeneous.

## Discussion

Prevention and vaccination account for a relatively minimal part of the healthcare spending in Western Europe, falling below 5% and 0.5%, respectively. This proportion has been found consistently across the 7 studied countries despite relatively heterogeneous data. We also found a downward trend over the study timeframe for most countries, with the exception of Sweden and England. These investment estimates should be considered in view of the number of diseases prevented and the far-reaching public health benefits of population-wide vaccination (i.e. a healthier population contributing to a healthier economy).^6^

To the best of our knowledge, this study is the first to systematically document and compare vaccine spending across Europe. Several limitations should be acknowledged. First, the OECD data did not allow us to appraise national spending specific to vaccines. The use of the OECD data, although incomplete, nonetheless offered some degree of standardization that facilitated comparisons across the selected European countries in terms of spending devoted to the prevention of communicable disease. Second, although national sources of spending data were sought, they were scarce and heterogeneous. Additionally, it has proven difficult to clearly identify what was actually included in the amounts disclosed. For instance, it was not possible to ensure that the reported amounts of money were effectively spent only on vaccine procurement or whether they included items such as vaccination awareness campaigns or other activities.

The portion of the healthcare spending allocated to vaccines should thus be reliably and systematically documented and/or made publicly accessible. When data are available, more details on cost items should be disclosed. In most countries, clearly identifying the cost components of prevention and vaccination spending is very difficult. Therefore, any comparison between countries should be made with caution.

The portion of healthcare spending allocated to prevention and vaccines is also somewhat lower than that assigned to medical technologies and devices. Recent reports from professional associations note that EU member states spend approximately 7.5% of their total healthcare expenditures on medical devices^7^ and 1% on *in vitro* diagnostic technologies.^8^

Vaccines have some specificities that make them probably more vulnerable to budget cuts: i) they are administered to healthy individuals (or at least to those who are free of the disease concerned and thus not seeking a cure); ii) the health impact of some of the diseases that they protect against, such as diphtheria, tetanus, poliomyelitis or pediatric meningitis of hemophilic influenza type B, appears to have been “forgotten” in Europe; and iii) in contrast to vaccine costs, their benefits are not necessarily observable in the short term.

Our study showed a declining trend in the portion of healthcare spending devoted to vaccines, although a growing number of diseases can be prevented by vaccines, which currently protect the general population of the studied countries from up to 19 bacterial or viral diseases. Several factors might explain such a trend: the decline in some vaccination coverage rates over time (e.g. influenza or measles-mumps-rubella vaccinations), the potential changes in the market competitive landscape for some vaccines (e.g., influenza) or the possible revisions of some national vaccination calendars (e.g. changes in dose regimen).Policymakers may also hold some misperceptions regarding the actual cost of vaccine procurement. Vaccination volumes are rather large, creating the impression that vaccine costs are high and volumes unrestrained. Nonetheless, the actual investment in vaccines is poorly documented. We addressed this gap and consistently showed that vaccine procurement does not constitute more than 0.5% of the healthcare spending in Western European countries.

Part of the healthcare spending currently allocated to vaccines should thus be preserved or even increased given their substantial public health benefits.^9^ Vaccines benefit other individuals in the population (through the so-called herd immunity effect) and thus help society as a whole. Furthermore, the span of vaccine protection might be broader because viral or bacterial infections can lead to a panel of multiple diseases. For instance, HPV (6,11,16 & 18) infection can cause not only cervical cancers but also genital warts, as well as vulvar, vaginal and anal cancers.

Policymakers should thus balance the level of investment in vaccines with the far-reaching benefits of vaccination, which protects the entire population and economy against potentially troublesome and resource-intensive outbreaks and prevents the resurgence of infectious diseases.^10-13^ According to multiple published cost-effectiveness analyses that compared vaccinations with no vaccinations, a lack of vaccination (or stopping vaccinations) could actually be much more expensive than vaccinations.^14-16^ Confronted with budget restrictions, policymakers may be tempted to seek immediate economic savings. However, to sustain universal healthcare systems, policymakers should carefully consider the broader interaction among economic, social and political sustainability issues.^17^

Spending on vaccinations should also be interpreted in view of the fairly high investment required to develop and safely produce vaccines, which are biological products intended for large-scale use in healthy populations. Vaccine production necessitates excellent control of variability in the living component and strict quality assurance of complex manufacturing processes.

Further studies are needed to devise alternative and/or mixed funding models to promote vaccination while contributing to the sustainability of healthcare systems. For instance, co-payment or private insurance schemes based on age, the degree of risk and the type of vaccination could be investigated. These new funding models should be designed with full consideration of fair and equitable access to healthcare and prevention.

Further methodological studies could also be envisaged, notably studies on how to enhance national vaccination calendars in view of evolving public health needs and budgetary constraints. In Western European countries, this optimization is supported by the need to ensure healthcare system sustainability as the population ages at an unprecedented pace. For this purpose, our analysis of vaccine spending could be complemented by the quantification of the health benefits and outcomes provided by national vaccination calendars.

In conclusion, vaccines constitute a minimal portion of national healthcare spending in Western Europe (≤0.5%). Our analysis has shown a net trend toward decreased spending in recent years, with the exception of Sweden and England. Nonetheless, national vaccination calendars may protect the population from up to 19 debilitating and potentially life-threatening diseases. Vaccination is thus a wise investment that absorbs a relatively low portion of national healthcare spending relative to its substantial benefits, which extend well beyond individual health and benefit the entire population and society.

In the current constrained budgetary context, vaccination budgets should be documented, preserved or even strengthened to sustain the population's health and to avoid longer-term health problems and costs. In addition, as the population ages, the development of vaccination programs with a life-course approach and sufficient coverage rates is recommended to promote healthy aging in Europe.

Future studies are encouraged to further enhance national vaccination calendars subject to budgetary constraints, demographic changes and public health priorities. Such efforts will necessitate the explicit consideration of population health outcomes that are achievable with vaccines as well as more granularities in the available budgetary data.

## Materials and methods

### Geographic scope

We selected 7 European countries: the 5 largest countries in terms of population, accounting for approximately 317 million (M) inhabitants in 2015 (Germany ≈81 M, UK ≈65 M, France ≈64 M, Italy ≈61 M and Spain ≈46 M), as well as 2 additional countries from northern and southern Europe whose populations amount to ≈10 M inhabitants (Sweden and Portugal). This panel of countries constituted a good mix of public vs. private markets and regionalized vs. centralized countries. This selection ensured strong representation of the different modalities of vaccine procurement across Western Europe.

### Healthcare, prevention and vaccine spending

We proceeded in 2 steps to document healthcare, prevention and vaccine spending in the selected countries. First, we queried the OECD online databases. Our search spanned from 2008 to the most recent data point available (typically 2012 or 2013) to estimate the portion of national total healthcare expenditures devoted to prevention and to vaccines.^18^ Total current healthcare expenditures (OECD code HCTOT), prevention and public health expenditures (OECD code HC6) and the prevention of communicable diseases expenditures (OECD code HC63) were extracted for the 7 selected countries. Data were available from 2008 with varying levels of completeness depending on the country. HCTOT expenditures were available for all countries, HC6 expenditures were available for all countries except the UK, and HC63 expenditures were available only for France, Spain and Sweden.

Because the OECD codification did not allow for specific quantification of the spending devoted to vaccines and/or vaccination, we also sought national healthcare, prevention and vaccine spending data (Table 3). Data were retrieved from various sources with different time horizons:

– Annual official statistics in France,^19^ Spain,^20^ Sweden,^21^ and Portugal.^22^ Data were available from 2008 to 2013 for France, from 2008 to 2012 for Spain and Portugal, and from 2011 to 2013 for Sweden. Because no specific vaccine spending estimates were available for Spain (only estimates for the prevention of communicable diseases, including vaccines), we used published estimates.^23^
– Health insurance databases in Germany.^24^ These databases included data from the Statutory Health Insurance (SHI) available through 2014. The SHI covers up to 90% of the German population and the remaining 10% are covered by private health plans.
– Official report from the House of Commons in England on National Health Service (NHS) spending^25^ and from the Department of Health in England on prevention spending.^26^ For vaccine spending, we identified 2 official reports commissioned by the Department of Health in England.^27,28^ Therefore, we only found data on NHS, prevention and vaccine spending for England (and not the UK) for 2006/07 and 2009/10.
– Official national report from the Medicines Utilization Monitoring Center of the Italian Medicines Agency^29^ and a report from advisors to the National Health System in Italy: The European House – Ambrosetti report.^30^ The latter is a think tank that aims to shape future healthcare scenarios and to develop proposals for change to improve the functioning of the Italian healthcare system and to allow for sustainable evolution over the long term. These two reports provided data for 2013 and 2014.

Table 4 provides a comparative overview of the prevention spending items included in each country's reports and in the OECD database, highlighting the heterogeneity across the different recording systems of the studied countries.

### Vaccine spending evolution

The evolution of vaccine spending over time was expressed in actuary terms using the compound annual growth rate (CAGR), which represents the constant year-to-year growth rate of an investment over a specific period of time.^31^ The CAGR between *t*_*0*_ and *t*_*n*_ is given by:

$$CAGR_{t_{0},t_{n}}={\left(\frac{V_{t_{n}}}{V_{t_{0}}}\right)}^{\frac{1}{t_{n}-t_{0}}}-1$$

where $V_{t_{0}}$ is the value at *t*_*0*_, $V_{t_{n}}$ is the value at *t*_*n*_, and the difference *t*_*n*_ - *t*_*0*_ represents the number of years.

### National vaccination calendar

To balance the vaccination spending estimates with the number of diseases prevented, we retrieved the most recent national vaccination calendars for each country (Table 3).^32-38^ We distinguished between men and women as well as between healthy individuals and individuals suffering from one or more underlying conditions because vaccination calendars typically recommend specific additional vaccinations for individuals with such conditions.

## List of abbreviations

*CAGR*: Compound Annual Growth Rate
*HPV*: Human Papillomavirus
*NHS*: National Health Service
*OECD*: Organization for Economic Co-operation and Development
*SHI*: Statutory Health Insurance
*UK*: United Kingdom
